# Interpreting and coding causal relationships for quality and safety using ICD-11

**DOI:** 10.1186/s12911-023-02363-5

**Published:** 2023-11-16

**Authors:** Jean-Marie Januel, Danielle A. Southern, William A. Ghali

**Affiliations:** 1 Department of Biomedical Informatics, Rouen University Hospital, 37 Boulevard Gambetta, Rouen, 76000 France; 2 Translational Innovation in Medicine and Complexity (TIMC) Laboratory, Deep Care research chair, Multidisciplinary Institute in Artificial Intelligence, Université Grenoble Alpes (UGA) and Centre National de Recherche Scientifique (CNRS), Grenoble, France; 3 Centre for Health Informatics, Cumming School of Medicine, University of Calgary, Calgary, Canada; 4 Department of Medicine, University of Calgary, Calgary, Canada

**Keywords:** Causation, International Classification of Diseases, Quality and safety, Adverse events, ICD-11

## Abstract

Many circumstances necessitate judgments regarding causation in health information systems, but these can be tricky in medicine and epidemiology. In this article, we reflect on what the ICD-11 Reference Guide provides on coding for causation and judging when relationships between clinical concepts are causal. Based on the use of different types of codes and the development of a new mechanism for coding potential causal relationships, the ICD-11 provides an in-depth transformation of coding expectations as compared to ICD-10. An essential part of the causal relationship interpretation relies on the presence of “connecting terms,” key elements in assessing the level of certainty regarding a potential relationship and how to proceed in coding a causal relationship using the new ICD-11 coding convention of postcoordination (i.e., clustering of codes). In addition, determining causation involves using documentation from healthcare providers, which is the foundation for coding health information. The coding guidelines and examples (taken from the quality and patient safety domain) presented in this article underline how new ICD-11 features and coding rules will enhance future health information systems and healthcare.

## Background

Determining causation is tricky in medicine and epidemiology. However, it is fundamental in decision-making [1–3]. Real-world data are potential sources of evidence, but because of the complexity of health systems, their use in addressing causation is challenging [4, 5].

In health information systems, many circumstances necessitate judgments regarding causation (e.g., death caused by a bullet wound, lactic acidosis caused by poisoning, an infection caused by failure of sterile precautions related to a nurse’s error). In particular, the establishment of causal relationships represents a triple conceptual and methodological challenge, including searching for evidence on the potential link between exposure and outcomes (e.g., when an infection occurs after a surgical procedure), considering situations where multiple factors may be causal (e.g., when both individual and organizational factors may be identified as associated or related to an adverse event), and assessing the likelihood of a potential causal relationship (e.g., which information and which rules should be used in interpreting information and establishing certainty about a given causal relationship) [6, 7].

Historically, the causal nature of observed associations has been analyzed using the Bradford Hill criteria (i.e., strength of effects, consistency, specificity, temporality, biological gradient, plausibility, coherence, experiment, analogy) [8–11]. In addition, causal relationships represent complex systems characterized by a number of potential interactions between multiple potential causes [10–12]. Interpreting causal relationships involving adverse events remains an important epidemiological challenge.

Under the World Health Organization (WHO) leadership, the development of ICD-11, the 11th revision of the International Classification of Diseases (ICD), is a significant advance in ICD structure, goals, and uses. The 10th revision (ICD-10) has historically been used to assess volume of healthcare activity in order to establish rates of hospital reimbursement as well as to assess morbidity, mortality, and quality of care. ICD-11 introduces new features that permit a better description of healthcare activity and patient safety events.

In this article, we reflect on what the ICD-11 Reference Guide provides on coding for causation and judging when relationships between clinical concepts are causal [13]. Using quality and patient safety examples, we explain how to apply the new coding convention of postcoordination to depict relationships between codes that are determined to be causal. The main objective of this article is to present and explain recommendations from the ICD-11 Reference Guide on how to proceed in interpreting and coding causal relationships using the new coding convention.

## Main text

### New ICD-11 convention for assessing causal relationships

Many circumstances necessitate judgments regarding causation, such as the following:

1. Death (e.g., caused by bullet wound),
2. Injury with external cause (e.g., lactic acidosis caused by poisoning),
3. Adverse events in healthcare caused by a known factor (e.g., infection caused by failure of sterile precautions related to a nurse’s error), and
4. Clinical manifestations caused by an underlying disease.

ICD-10 has mechanisms for depicting causal relationships between diagnosis codes. These include implicit causal rules around the use of the chapter on external causes of injury, the dagger-asterisk system [14], and the mortality coding rules for underlying cause of death. Now, ICD-11 offers significantly enhanced mechanisms, both within the code structure itself (clustering, new quality and safety codes, extension codes) and in the Reference Guide, where detailed information is provided to guide coders in making judgments about causation. In addition, by better considering causal relationships and inferences in these causal relationships, ICD-11 allows coding of more complex situations with more precision.

The ICD-11 Reference Guide has information regarding connecting terms to determine cause of death [15], and another article in this series details the rules for clinical manifestations caused by an underlying disease [16]. However, the focus of this article is how to apply the new coding convention of postcoordination to represent the three-part model of quality and patient safety (developed by the WHO Family of International Classifications Quality and Safety Topic Advisory Group [17–19]) that is explained in detail in two other papers in this series [16, 20]. This postcoordination requires building code clusters, where a combination of codes can represent one clinical situation. The term “cluster” is an informal expression related to the novel ICD-11 postcoordination feature presented in detail in another paper in this series [21]. Briefly, postcoordination is a feature that, among other things, permits coders to combine causally interrelated clinical concepts into a single expression (e.g., intracranial hemorrhage due to an overdose of warfarin—8B0Z/PL00&XM86W0/PL13.0). A related core feature of ICD-11 is the availability of extension codes (i.e., supplementary codes that capture more granular details, such as the role of warfarin XM86W0 in the preceding example.

Quality and patient safety provide some of the best examples of assessing causal relationships in clinical situations, hospitals and the community, and ambulatory care settings. Based on the new coding convention of clustering, ICD-11 allows for the formalization of links that characterize causal relationships using postcoordination, establishing a mechanism that explains how a potential cause is the real cause of harm. In detail, quality and patient safety harm is usually represented by a standard ICD-11 diagnosis code from almost any chapter of the classification. In addition, special chapters (Chapter 22: Injury, poisoning or certain other consequences of external causes; Chapter 23: External causes of morbidity or mortality) list sets of harms and injuries with possible causes such as substances (e.g., drugs and medicaments), procedures, devices, and other healthcare-related causes like problems with transfusions or missed or incorrect diagnosis. In addition, Chapter 23 provides a list of codes (PL11.x, PL12.x, PL13.x, and PL14.x) for identifying modes (i.e., mechanisms to determine the relationship between potential causes and harm). Postcoordination is used to develop algorithms for coding harm, cause(s), and mode(s)— the three parts in the three-part model mentioned earlier—and the resulting cluster of codes represents the final description of a given clinical situation with a potential causal relationship. Accordingly, a unique clinical situation of quality and patient safety is identified by the harm, which is contextualized by one or more potential causes and modes.

### Method for considering causation with ICD-11

Determining causation for ICD-11 coding through what is written in medical charts is based on the following:

- use of the medical chart as the foundation for coding health information;
- interpretation of documentation from healthcare providers as the source information, which includes
  - ◦ juxtaposition and exploration of clinical concepts in raising the possibility of causal relationships (e.g., A beside B, A before B, A after B, A unrelated to B, A associated with B, A caused by B, A causing B) and
  - ◦ review of the presence of connecting terms as key elements in assessing the level of certainty about a potential relationship between A and B.

### Clinical concepts for potential causation

**Fig. 1:**
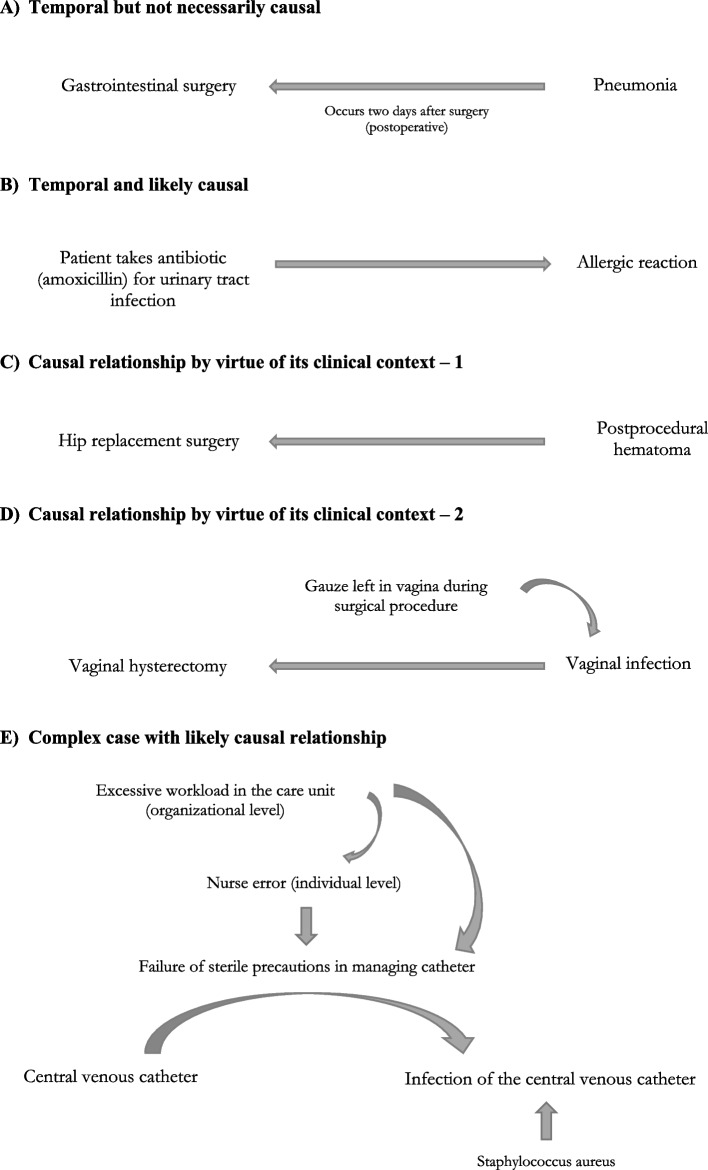
Examples of situations where causal relationships are contemplated

As described in a previous paper in this journal issue [19], the three-part model for describing healthcare-related adverse events in ICD-11 was developed for the purpose of standardization. Implicit in its use is the assumption that a diagnosis is causally related to a healthcare factor (i.e., relationships between harm, cause, and mechanism are assessed to link harm to a cause such as medication, surgical procedure, medical device, or other aspects of care). According to the Reference Guide, causal relationships can exist between any two conditions regardless of when each condition was reported in the course of the patient stay in hospital. However, to invoke the three-part model, there needs to be judgment regarding a causal link to determine the underlying cause of a harm diagnosis. Five examples are presented in Fig. 1. These illustrate the potential difficulties and possibilities in interpreting causal relationships linking harm to a cause.

The first two examples are based on the temporal sequence of events. In the first one (Fig. 1A), causation is based only on temporal terms. The mention of pneumonia occurring two days after the surgical intervention alone does not allow the coder to be certain of the causal relationship between surgery and infection. In contrast, the second example (Fig. 1B) suggests more certainty in attributing anaphylactic reaction to the drug taken by the patient. In this situation, the temporality may provide evidence of a direct causal relationship between the drug and the adverse effect of the drug because the clinical experience provides well-known evidence about the risk of allergic reaction to the use of amoxicillin.

The following two examples (Fig. 1C, D) outline situations where the nature of the relationships is causal by virtue of their clinical context (i.e., postprocedural bleeding or hematoma, surgical site infection, postoperative wound dehiscence). The first involves a patient whose hip replacement surgery was followed by the development of a hematoma (Fig. 1C). The second one describes a patient developing a vaginal infection several days after having a vaginal hysterectomy (Fig. 1D). It is noted that gauze had been forgotten in the patient’s vagina during the surgical procedure. In these examples, the injury or harm occurred after the surgical procedure, but it is mainly the clinical context that provides evidence of causal relationships.

Determining causation may be tricky when situations are more complex and include interactions between numerous factors with endogenous and exogeneous potential effects. As an example, Fig. 1E suggests numerous potential factors interacting to produce a central venous catheter infection. In this fifth example, the infection of a central venous catheter may be caused by three factors (direct or indirect): a failure of sterile precautions in managing the catheter that may be directly attributed to an error by healthcare worker (i.e., the operator) and could be the indirect consequence of an excessive workload in the care unit (suggesting a failure in the organization of the unit). In this complex situation, the excessive workload could have potential interactions with the operator’s failure to execute sterile precautions and indirectly with the central venous catheter infection. This suggests that interpretation of the causal relationship may be complex—that is, resulting from several potential factors that interact with the others, together providing the complete causal relation for the central venous catheter infection.

### Coding examples involving judgments on causation

According to the ICD-11 Reference Guide, causal relationships can exist between any two or more conditions regardless of when each condition was reported in the course of the patient’s stay in hospital. A key new feature of ICD-11 compared to ICD-10 is its ability to make links, including causal links, between two or more conditions and/or modalities that interact with the other(s), regardless of whether the three-part model is used. Based on the new coding rules in ICD-11, causal relationships can be coded using combinations of two types of codes: stem codes (Reference Guide 2.9) and extension codes (Reference Guide 2.10).

Stem codes may be entities or groupings of high relevance or clinical entities that should always be described as one entity. These codes are organized in 26 chapters that follow the typical pattern of the ICD, relating to etiology, relevant organ system, maternal and perinatal status, external causes (of death or complications), and factors that may influence health status. Extension codes are lists of additional information that can be added to stem codes to give more details about and contextualize a clinical situation. There are two main types of extension codes:

- type 1 extension codes, allowing detail to be added to a stem code in terms of severity, temporality, etiology, topology, specific anatomic detail, histopathology, dimension of injury, dimension of external causes, consciousness, substances and medications, and medical devices
- type 2 extension codes, allowing detail to be added to a diagnosis, which may indicate how the diagnosis is to be used and/or interpreted regarding discharge diagnosis types, diagnosis timing in relation to surgical procedure, diagnosis method of confirmation, diagnosis certainty, obstetrical diagnosis timing, and capacity or context

Extension codes are exclusively used to provide supplementary information on a linked stem code; accordingly, extension codes cannot be used alone. By combining stem codes, or stem codes and extension codes, ICD-11 allows coding of different clinical concepts, and the capture of causal relationships between two or more conditions is possible. The development enables multiple codes to be used together as a code cluster by combining them using either a forward slash (/) for separation of stem code concepts or an ampersand (&) to link extension codes to a linked stem code. More details on postcoordination and combining codes are found in our series paper describing the ICD-11 postcoordination feature [21]. This new function allows for the rich description of complex clinical concepts. In addition, combinations of stem codes and extension codes are valid for use with or without the three-part model. The three-part model is useful when conditions for harm, cause, and mode are all needed to interpret causal relationships. In the absence of clear information in assessing the certainty of causal relationships (i.e., the absence of specific words and failure to check with the documenting clinician), the recommendation is to code each condition separately and not link conditions in a cluster (Reference Guide 2.24.5).

Based on the five clinical examples from the previous section displayed in Fig. 1, in this section we develop different ways to code causal relationships according to the Reference Guide. The first example (corresponding to the clinical concept represented in Fig. 1A) concerns a temporal but not necessarily causal relationship. In this example, the patient got pneumonia (without a specified organism) two days after gastrointestinal surgery. The potential link between A, gastrointestinal surgery as the potential cause of injury or harm, and B, pneumonia as the injury, is based only on the temporality between both, the surgery occurring two days before pneumonia, which is not enough to interpret a causal relationship between them. Here, the mode, which would provide for how gastrointestinal surgery could be determined to be the true cause of pneumonia, is not available. The code PL11.Z is not applied because the documentation does not mention any specific mode or mechanism by which pneumonia occurred, except for the temporality captured by the extension code XY7V. Accordingly, we are not certain about the causal relationship in this clinical situation. We suggest coding it as follows:

- *Harm: Pneumonia* (stem code 1: CA40.Z)
  - Diagnosis (of harm) timing: Postoperative (extension code 1: XY7V)
- *Cluster:* CA40.Z&XY7V

The second example presents a patient who suffers anaphylaxis after amoxicillin administration (Fig. 1B). In this case, the harm is defined by the stem code 4A84.1, “Drug-induced anaphylaxis,” and the cause is identified by the stem code PL00, “Drugs, medicaments or biological substances associated with injury or harm in therapeutic use.” Again, postcoordination coding rules may be used in this case, using the extension code for amoxicillin, XM7CM1, giving precision about the drug that caused the anaphylactic reaction:

- *Harm:* Drug-induced anaphylaxis (stem code 1: 4A84.1)
- *Cause:* Drugs, medicaments or biological substances associated with injury or harm in therapeutic use (stem code 2: PL00)
  - *Specific medication*: amoxicillin (extension code 1: XM7CM1)
- *Cluster:* 4A84.1/PL00&XM7CM1

However, we can do even better and indicate whether the anaphylactic reaction was avoidable or not, depending on whether the patient had a known allergy. If yes, we use PL13.6 for “Medication or substance that is known to be an allergen, as mode of injury or harm.” If unknown, we would use PL13.2 for “Drug-related injury or harm in the context of correct administration or dosage, as mode of injury or harm” (medication was used correctly, but the patient reacted to it). Accordingly, precision may be added about the potentially avoidable nature of the cause using extension codes PL13.6 or PL13.2 as follows:

- *Harm:* Drug-induced anaphylaxis (stem code 1: 4A84.1)
- *Cause:* Drugs, medicaments or biological substances associated with injury or harm in therapeutic use (stem code 2: PL00)
  - *Specific medication: *amoxicillin (extension code 1: XM7CM1)
  - *Precision about the cause:* Medication or substance that is known to be an allergen, as mode of injury or harm (stem code 3: PL13.6)
  - Drug-related injury or harm in the context of correct administration or dosage, as mode of injury or harm (stem code 3: PL13.2)
- *Cluster:* 4A84.1/PL00&XM7CM1/PL13.6 or 4A84.1/PL00&XM7CM1/PL13.2

In the third and fourth examples (Fig. 1C, D), the causal relationship is determined by virtue of the clinical context; the clinical situation naturally implies a causal relationship. In the example from Fig. 1C, the nature of the surgical intervention—that is, a large and deep incision at the hip area—suggests that the cauterization of some small vessels failed during the procedure. In this case, the three-part model is unnecessary because the harm and cause conditions are sufficient to assess the causal relationship between the hematoma (the harm) and hip replacement surgery with an open approach (the cause).

- *Harm: *Haemorrhage or haematoma complicating a procedure, not elsewhere classified (stem code 1: NE91.0)
- *Cause: Musculoskeletal procedure associated with injury or harm, open approach* (stem code 2: PK80.80)
- *Cluster: *NE91.0**/**PK80.80

In the example from Fig. 1D, the mode (i.e., gauze left in the vagina during the surgical procedure) may be naturally interpreted as the principal factor in the infection. In this case, the three-part model is needed to assess the causal relationship between the infection and the surgical procedure.

- *Harm: *Surgical site infection (stem code 1: NE81.2)
- *Cause: *Gynaecological or breast procedure associated with injury or harm in therapeutic use, per orifice approach (stem code 2: PK80.53)
- *Mode: *Foreign body accidentally left in body, as mode of injury or harm (stem code 3: PL11.3)
- *Cluster: *NE81.2**/**PK80.53**/**PL11.3

In the last example, shown in Fig. 1E, the causal relationship is more complex than in the previous four examples because of numerous potential factors and interactions between them. Nurse (operator) error is directly responsible for the failure of sterile precautions in managing the catheter, so it is the principal explanation for central venous catheter infection. Further, the failure of sterile precautions and the nurse error at the individual level could also be influenced by an excessive workload in the care unit, an organizational misfunction that compromised healthcare performance. Finally, while there is no specific ICD-11 code for provider performance compromised by excessive workload, a more general code can be used to describe this situation (namely, “Other specified aspects of care associated with injury or harm”).

- *Harm: *Infection arising from device, implant or graft, not elsewhere classified (stem code 1: NE83.1)
  - *Infectious agent*: Staphylococcus aureus (extension code 1: XN6BM)
- *Mode 1: Failure of sterile precautions, as mode of injury or harm* (stem code 2: PL11.4)
- *Mode 2: Operator error, as mode of injury or harm* (stem code 3: PL12.5)
- *Mode 3: Provider performance compromised by excessive workload* (stem code 4: PL14.Y Other specified aspects of care associated with injury or harm)
- *Cluster:* NE83.1&XN6BM/PL11.4/PL12.5/PL14.Y

### Assessing certainty in causal relationships

The most important challenge in assessing and then coding causal relationships is undoubtedly suggesting (assessing) potential causal relationships when possible causes are numerous and establishing the reality of those in clinical situations using medical chart documentation—that is, determining what is a clear causal relationship, what is a possible causal relationship (i.e., the causal relationship is not clearly established), and what is not a causal relationship. While the previous section was essential because it explained how to proceed in coding causal relationships in several types of clinical situations, this section is potentially even more essential because it aims to explain how to establish a level of certainty about causal relationships in consideration of the complexity of healthcare documentation.

The assessment of connecting terms in the medical chart is fundamental to applying the new convention for coding causal relationships with ICD-11. The Reference Guide provides a list of words (i.e., connecting terms) that coders should look for when assessing medical records. These are listed in Table 1. The connecting terms are categorized into three classes according to decreasing level of certainty in interpreting causal relationships. In particular, coders should first look for some specific words that naturally imply certainty of causality, such as “due to,” “caused by,” or “arising from.” Terms such as “associated with” and “incidental to” provide unclear information about causal relationships. On the other hand, events may be documented using connecting terms that add even more ambiguity for the coding expert, such as “with,” “after,” “in,” and “following,” for example. These terms are temporal descriptors that in and of themselves are insufficient to infer causation. In situations where these connecting terms (i.e., column 3 of Table 1) are used, the Reference Guide formally suggests that selected codes simply reflect temporal relationships, not causal relationships. In the example from Fig. 1A, where pneumonia occurred two days after surgical intervention, it is not possible to determine the causal relationship between surgical intervention and pneumonia based only on the word “after,” which indicates the temporality of harm in relation to the surgical procedure. This example demonstrates that temporality is not the same as causality. In this case, coders need to find additional terms or ancillary information or contact the documenting clinician for further guidance and more details to establish a causal relationship.

**Table 1:** List of terms implying a causal relationship from the ICD-11 Reference Guide

Terms implying a clear causal relationship	Terms where the causal relationship is unclear	Terms not implying causal relationship
as (a) complication of, complicated by, complicating, complication(s) of as a cause of, cause of, caused, caused by, causing as a result of, resulted in, resulting in, with resultant, with resulting because of due to from induced, induced by leading to, led to related to, precipitated by producing secondary to likely related to^a^ possibly secondary to, probably secondary to^a^ may be the reason for^a^	associated with accompanied by incidental to	after also and during with arising in or during consistent with followed by, following incurred after/during/in/when occurred after/during/in/when/while postoperatively, postoperative, occurred post-op^b^

^a^Coding judgment call. However, the clinician is making a causal inference with this term

^b^Terms like “postoperative,” “post-op,” “postprocedural,” etc. are a special situation because these have historically been considered and in some coding systems are considered to be indicative of a causal link. However, conditions such as urinary tract infection, pneumonia, and atrial fibrillation may temporally arise after surgery, without necessarily being caused by surgery. For this reason, in such cases it is necessary to look for more explicit causal connections in establishing inferences about causation using ICD-11

In some other clinical situations, there is no need to identify connecting terms or modes in establishing the link between the cause and the harm because the causal relationship is naturally clear by virtue of the clinical context. These clinical situations are characterized by a specific context in which harm would not have occurred in the absence of a procedure or a device problem. These particular situations include postprocedural bleeding or wound hematoma, postoperative wound infection or dehiscence, and drug eruption (drug rash), among others. Examples of this are seen in Fig. 1C, D. In the same way, there are also some clinical situations where it is possible to use individual codes, without any clustering to establish causal relationships. In such situations, only one code called the precoordinated code is needed to capture both the harm and its causation by a procedure (e.g., GC72 for “Postprocedural urethral stricture” or GC70 for “Postoperative adhesions of vagina”). In other clinical situations where harm occurs, such as postoperative pneumonia (Fig. 1A), postoperative pulmonary embolism, or postoperative atrial fibrillation, the harm is not necessarily caused by the surgical procedure.

There are some clinical situations where problems arise but without adverse consequences to the patient (e.g., a patient falls in the healthcare setting but is not injured, a drug is administered to the wrong patient but does not harm this patient). In such clinical situations, the medical chart contains no documentation about potential harm to the patient in relation to the problem. In such clinical situations, coders should be use codes from Chapter 24: Factors influencing health status or contact with health services, in the section of codes entitled “Health care-related circumstances influencing the episode of care, without documented injury or harm.” This is different from Chapter 23: External causes of morbidity or mortality, given that Chapter 24 describes clinical situations where no harm to the patient is caused.

### Need for relevant and valid healthcare documentation

In coding potential causal relationships and inferences about causation, reliable and valid healthcare documentation is a prerequisite. Yet the quality of clinical documentation in charts varies across countries, institutions, and even across clinical services and units within institutions. Furthermore, even when clinical documentation is extensive and highly detailed, there are many instances where causal relationships are unknown and unclear. Lastly, in situations where documentation is robust and causation is clear, there is still the inherent challenge of ensuring that coders are trained on the nuances described and discussed in this article and the ICD-11 Reference Guide. For the latter, the WHO is developing multifaceted educational materials for coding.

Our emphasis in this article is on how to consider and represent causal relationships when using ICD-11 to code healthcare-related events. With that emphasis, we have not explicitly discussed the implication of these new ICD-11 features in relation to whether human or data analysis systems can correctly interpret a coder’s intention to document a causal association in a code cluster. Fortunately, the framing of Chapter 23 and its entire content is a mitigating factor; any code cluster involving a diagnosis code selected by a coder from Chapter 23 is explicitly stating that the item from that chapter caused the linked diagnosis in the cluster. Nevertheless, coders may wish to create diagnosis code clusters that do not involve Chapter 23 while still intending to indicate causal associations. Future ICD-11 field testing and analytic studies are needed to assess the extent to which coder intention will be unambiguously represented in the ICD postcoordination system.

There is no question that the ICD-11 postcoordination and extension code features have exponentially increased the complexity of the ICD-11 system. Yet, in doing so, these features have also exponentially increased the clinical richness and power of ICD-11. These parallel truths highlight the challenge and opportunity ahead as ICD-11 implementation unfolds globally.

## Conclusions

ICD-11 has several new features that enable a richer description of clinical situations and combinations of diagnoses. The causal relationship between clinical concepts is always a matter of interest in healthcare documentation because health and disease are affected by factors that are often causal contributors to a health state. The coding features in ICD-11 that enable better capture of causal relationships include clustering functions, a number of “code also” rules, the external causes chapter (Chapter 23), and many of the codes in the extension code chapter (Chapter X). In addition, the coding guidelines relating to connecting terms and the clinical examples presented in this article underline how new ICD-11 features and coding rules will enhance future health information systems.

## Data Availability

Not applicable.

## Abbreviations

ICD: International Classification of Diseases
ICD-9-CM: International Classification of Diseases, 9th revision, clinical modification used in the United States
ICD-10: International Classification of Diseases, 10th revision
ICD-11: International Classification of Diseases, 11th revision
